# BELTracker: evidence sentence retrieval for BEL statements

**DOI:** 10.1093/database/baw079

**Published:** 2016-05-12

**Authors:** Majid Rastegar-Mojarad, Ravikumar Komandur Elayavilli, Hongfang Liu

**Affiliations:** ^1^Department of Health Sciences Research, Mayo Clinic, USA; ^2^University of Wisconsin-Milwaukee, Milwaukee, WI, USA

## Abstract

Biological expression language (BEL) is one of the main formal representation models of biological networks. The primary source of information for curating biological networks in BEL representation has been literature. It remains a challenge to identify relevant articles and the corresponding evidence statements for curating and validating BEL statements. In this paper, we describe BELTracker, a tool used to retrieve and rank evidence sentences from PubMed abstracts and full-text articles for a given BEL statement (per the 2015 task requirements of BioCreative V BEL Task). The system is comprised of three main components, (i) translation of a given BEL statement to an information retrieval (IR) query, (ii) retrieval of relevant PubMed citations and (iii) finding and ranking the evidence sentences in those citations. BELTracker uses a combination of multiple approaches based on traditional IR, machine learning, and heuristics to accomplish the task. The system identified and ranked at least one fully relevant evidence sentence in the top 10 retrieved sentences for 72 out of 97 BEL statements in the test set. BELTracker achieved a precision of 0.392, 0.532 and 0.615 when evaluated with three criteria, namely full, relaxed and context criteria, respectively, by the task organizers. Our team at Mayo Clinic was the only participant in this task. BELTracker is available as a RESTful API and is available for public use.

**Database URL:** http://www.openbionlp.org:8080/BelTracker/finder/Given_BEL_Statement

## Introduction

Biological networks are powerful and expressive means of representing biological information and knowledge (1). Biomedical literature has been the primary source of information for curating biological networks. Domain experts, based on manual review of the literature, primarily carry out the process of curation of biological networks. This effort involves a vast amount of time and often leads to a substantial lag in formalizing the information in the literature to formal representation.

There have been considerable efforts made by domain experts to design formal representation standards to allow for a seamless transition from the natural language texts to structured representation without any loss of information. Biological Expression Language (BEL) (2) and System Biology Markup Language (SBML) (3) are two of the existing representation methods that effectively express the semantic of biological pathway events. These representation models can support many downstream computational applications (1). There are very few text-mining efforts to automatically extract biological network information in scientific literature to such formal representation standards. Identifying the relevant sentences in the ever-growing body of scientific literature that best describe the biological pathway event is the first and most critical step in the above process.

At the right juncture, the BioCreative V challenge has been organized, with a goal of addressing the dearth of tools to formalize information in natural language to BEL representation in a limited context of causal relationships. The organizers defined the following two tasks, one centered on information retrieval and the other on information extraction (4, 5). The two tasks are:

1. Given textual evidence for a BEL statement, generate the corresponding BEL statement (information extraction [IE] task)
2. Given a BEL statement, provide, at most, 10 evidence sentences (information retrieval [IR] task)

We participated in both tasks (6, 7). In this article, we describe our approach to extract, at most, 10 evidence statements from a given a BEL statement, which is more of an information retrieval–centric task. We describe in detail the BELTracker tool that we developed for the BioCreative V task in the sections below.

The paper is organized as follows: First, we briefly review related work, where we discuss the gaps in the current state-of-the-art tools and cover how they are unsuitable for the BioCreative V task. Second, we briefly describe the BEL framework. Third, we explain our approach and present the results of the system on the test dataset, which was evaluated manually by the organizers. Finally, we conclude the paper with a brief discussion on the performance of the system against the test data, its limitations, and possible future directions of the current work.

### Related work

Biological networks and pathways contain rich semantic information on various cellular processes and how they determine various physiological and pathological conditions of an organism. Despite various collaborative efforts, there is considerable lag in the curation of biological pathways from the biomedical literature. It takes a substantial amount of time for the experts to manually review the literature and infer the right information (8, 9). There are two distinct stages in the curation process: (i) retrieving the relevant literature pool regarding events of a biological pathway involving proteins, processes, etc and (ii) identifying only the relevant sentences from the entire article and identifying the exact biological event from the sentences. Text mining, to some extent, can partially help in this curation process. While IR systems can play a critical role in identifying the relevant literature pool and retrieving evidence sentences for biological events from scientific literature, IE systems could assist with the latter steps. Typically, IE systems identify biological entities and extract relationships between them from the literature. They then help synthesize pieces of information to generate biological networks. There have been numerous prior efforts directed at addressing both stages in the biomedical domain. Here, we briefly review some of the relevant IR and IE systems that may play an important role in automating the process of curating biological networks.

In the information retrieval paradigm, systems broadly fall into three categories: (i) keyword-based retrieval, (ii) concept-based retrieval and (iii) IE-based retrieval. PubMed (http://www.ncbi.nlm.nih.gov/pubmed) and iHOP (10) belong to the first category, though PubMed retrieves only abstracts, while iHOP retrieves even the sentences that match the query based on co-occurrences. PubMed also attempts to retrieve documents based on human-indexed MeSH terms to further improve accuracy. iHOP builds on the platform of PubMed by identifying the co-occurrence of the terms that occur in the query. The subsequent versions of iHOP further evolve toward identifying the entities, especially genes, and attempting to build gene networks based on sentence level co-occurrence. While it will be more useful to retrieve evidence sentences based on entities when compared with keyword-only searches, the effectiveness of iHOP is largely limited given its focus only on identifying genes within the sentences.

Chilibot, another retrieval system based on user-defined entities, is an attempt to indicate a moderate advancement in the paradigm of IR from keyword-based retrieval to entity-based retrieval (11). Chillibot is partially an attempt to synthesize the features in PubMed and iHOP. It presents a graphical representation of the relationships, retrieved from biomedical literature, among user-provided entities. This system allows users to enter, at most, two biological entities and generates a network using those entities and retrieved relations (biological entities as nodes and relations as edges). Unlike iHOP, Chilibot extraction of relationships from sentences is not based on a simple co-occurrence of entities within a single sentence. Instead, it uses simple linguistic rules to uncover links between the entities mentioned within a single sentence. Chillibot also predominantly generates gene networks like iHOP.

On the commercial end, there were other similar efforts made to extract biological relations and networks from literature. PathwayAssist (12) captures pathway information in a relational database, ably assisted by MedScan, a text-mining tool that extracts biological entities and relationships from the literature. PathwayAssist is a concept-based IR system and marks a considerable advancement over other entity-based IR.

Typically, biological events involve multiple events that go beyond simple entities such as a change in the molecular state, transport of molecules across cellular compartments (involving spatial dimension), and change in the cellular processes. The first-generation tools described above did not pay attention to any concepts beyond simple entities. GoPubMed (13) is an information-retrieval engine that truly extends beyond the notion of simple entities. While it may not have sophisticated features, such as generating biological networks, the recognition of broad concepts such as biological processes and molecular function outlined in gene ontology (14) is definitely a substantial advancement over all the other tools discussed above. GoPubMed’s ability to identify diverse biological concepts with a fair degree of accuracy helped lay the platform for the next generation of tools. The third-generation tools attempted to combine the best of the breed features of highly matured first-generation tools, such as PathwayAssist, and second-generation tools, such as GoPubMed, to develop information extraction– centered IR tools.

Ohta et al (15) developed a IR tool called the MedIE, a semantic search engine that allows users to query on stored triplets, namely *subject-verb-object* (SVO), where *subject* and *object* are biomedical entities and *verb* indicates relation type, such as: *activate, induce,* and *cause.* MedIE is a considerable advancement over the earlier-generation tools in the following ways: (i) It uses deeper syntactic analysis to extract relationships from the biomedical literature, and (ii) It uses a triple-store representation (SVO triplets) for indexing. MedIE opened avenues to query documents based on the preindexed metadata extracted from the documents, in addition to paving a way for IR to mature to handle sophisticated causal relation queries. To some extent, the IR paradigm of MedIE is similar to the SPARQL query directed against semantic triple stores.

None of these systems, however, can handle complex queries enriched with biological semantics. As part of BioCreative V shared tasks, one such task was organized, wherein the query in the form of BEL statements contains multiple components, each of them having semantic meaning. The task involves identifying the relevant evidence sentences from the biological literature, given a BEL statement. This task can be compared to a complex SQL query, with multiple *where* clauses directed against a natural language text. A typical BEL query, as outlined earlier and in the following sections, consists of entities, functions, and relationships involving various ontological classes. In the past, several challenges, such as Genomic TREC (16), Chemical TREC (17) and answering the CLEF Question (18) were organized to improve the state-of-the-art IR in the biomedical and clinical domain. Track 4 of BioCreative V is a first of its kind that involves sophisticated IR for queries loaded with complex semantic information.

In this article, we address three important challenges: (i) parsing a complex semantic query such as BEL and translating it into a simple keyword/concept retrieval without any loss in the semantics, (ii) identifying the relevant literature that best describes the semantics of the query and (iii) ranking the retrieved sentences in the order of relevance to the query.

## Biological expression language

In this section, we provide a brief background on the BEL statement. Fluck *et al.* (1) has outlined a detailed description of the BioCreative V BEL task and the BEL framework. BEL is currently an open-source initiative, initially incubated by Selventa (erstwhile Genstruct) (19). BEL statements denote causal and correlative links between biological entities that have an inherent capability to express relationships at different levels of granularity (1). Their expressive capabilities range from representing simple protein-protein links to relationships between biological processes (1). BEL statements are semantic triples of *subject*, *predicate*, and *object,* where *subject* and *object* are biological entities or another BEL statement, and *predicate* qualifies the relation between *subject* and *object*. So far, scientific experts have curated >180 000 BEL statements based on supporting evidence sentences from biomedical literature. These curation efforts were part of a broader initiative for curating large biological networks. To date, curation of 50 such networks have been completed.

Table 1 shows two evidence sentences and the corresponding BEL statements relevant to these sentences curated by the experts. Table 1 also highlights the various components of BEL statements listed below:

**Table 1:** Sample BEL curation from evidence sentences

Sentences	BEL statement
We showed that HSF 1 is phosphorylated by the protein kinase RSK2 in vitro we demonstrate that RSK2 slightly represses activation of HSF1 in vivo	1: kin (p (HGNC: RPS6KA3)) increases p (HGNC: HSF1, pmod (P))2: kin (p (HGNC: RPS6KA3)) decreases tscript (p (HGNC: HSF1))
Whereas exposure of neutrophils to LPS or TNF-a resulted in increased levels of the transcriptionally active serine 133-phosphorylated form of CREB	p(MGI: TNF) increases p (MGI: CREB1, pmod (P, S, 133))
BEL Elements: Relationship, Function, Entity, Namespace, Sequence position	

Table 1 shows example BEL statements curated from evidence sentences. Components of a BEL statement are highlighted using different colors.

- **Entity:** Biological entities such as gene, chemical, protein, etc.
- **Namespace:** The ontology that biological entities come from. The dataset contains entities from these ontologies: Mouse Genome Informatics (MGI) (20), Chemical Entities of Biological Interest (ChEBI) (21), Gene Ontology (GOBP) (14), Medical Subject Headings (MeSH) (22) and HUGO Gene Nomenclature Committee (HGNC) (23). The purpose of this element is to provide a reference to the entities.
- **Relation type:** Type of link between subject and object. There are four relationship types in the provided dataset: increases, decreases, directly increases, and directly decreases.
- **Function:** There are five categories of BEL functions: abundance, modifications, activities, processes and transformations.
- **Arguments for functions:** BEL functions can have up to three arguments, depending on its type. For example, the function protein modification (pmod) can have three arguments: (i) type of modification, (ii) modified residue and (iii) sequence position of the modification.

## Method

BELTracker has three main components:

- **Query translation:** Translating a given BEL statement into a query
- **Retrieval:** Retrieving the relevant citations from PubMed abstracts and full-text articles from PubMed Central (PMC)
- **Ranking:** Identifying the appropriate evidence sentences (at most 10) from citations and ranking their relevance to the given BEL statement

**Figure 1.**
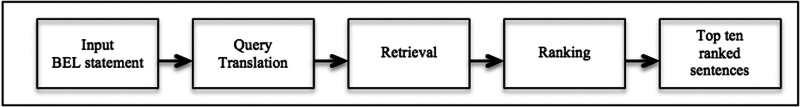
Overall workflow of BELTracker.

Figure 1 illustrates the overall workflow of the BELTracker system.

We implemented BELTracker in Java. We used several additional tools and resources, including ElasticSearch (https://www.elastic.co/), Weka (24), LibSVM (25) and Stanford Core NLP (26) as components of BELTracker.

### Query translation component

**Figure 2.**
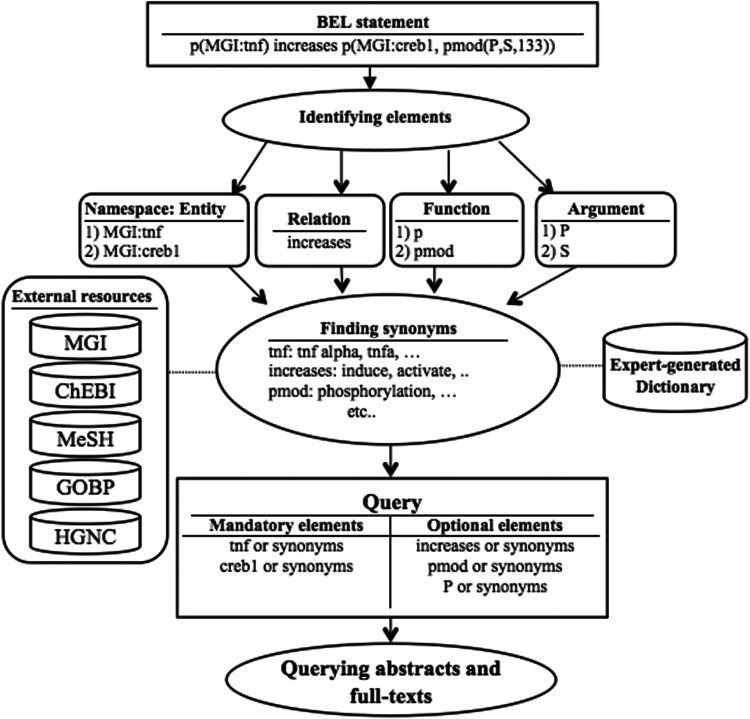
The query translation component.

Figure 2 illustrates the broad functionality of the query translation component.

As an initial step, BELTracker translates the given BEL statement into a query suitable to retrieve information from natural language indexed in a search engine. First, we identify the individual components in a given BEL statement (e.g. entity, function, and relation) highlighted in Table 1. Each component of the BEL statement finds representation in the query. We further supplement the query by adding synonyms for certain elements, such as entities. For query expansion, we rely on both externally curated and internally developed resources, depending upon the component. For the synonyms for entities, we use several available standard knowledge resources, including HGNC, MGI, Entrez, and Gene ontology. For expanding words that describe relationships, we create a list of synonyms for each relation type based on domain experts’ knowledge. For example, the relation keyword *increases* will be expanded to words such as *induce* and *activate* that best characterize the relation often used by authors to describe *increase*. Similarly, we also have an expert-curated dictionary of synonyms for BEL functions and their arguments.

However, we distinguish the source for expansion by assigning different weights to the individual components of a query. We assign greater importance to externally curated standard resources widely used by the community against the internally curated resources. Hence, BELTracker assigns higher weights to entities when compared to functions and relations. We assigned lesser weights to internally generated resources due to their limited vocabulary. As a final step in query translation, we combine the expanded query elements with appropriate Boolean logic, making some mandatory (by using *AND*) and others optional (by using *OR*). The presence of entities or their synonyms in the retrieved citations is mandatory, while the presence of other elements, such as functions and relationships, are optional. Similarly, synonyms were assigned lower weights when compared with the original entities that occurred in the BEL statement. BELTracker currently assigns the weight in an ad-hoc manner, namely assigning a weight score of two to the names expressed in the BEL statement, while it assigns a weight score of one to their synonyms.

Assignment of differential weights was possible due to our choice of ElasticSearch as the search engine to index and retrieve relevant citations. We take advantage of the inherent *boost* features of the search engine by assigning differential weights to every element of the query.

### Retrieval component

To retrieve relevant citations for the translated BEL query, we first created an index of all PubMed abstracts and PMC full-text articles. The index consists of 24 million PubMed abstracts and 3.8 million full-text articles. We downloaded the XML file format of both PubMed abstracts and full-text articles and indexed them using ElasticSearch. The query generated by the previous component (described in Query translation component Section) is used to query against the ElasticSearch index to retrieve the most relevant citations (up to 1000 from abstracts and 1000 from full texts). The ElasticSearch assigns relevant scores to each citation. BELTracker uses this score to rank the citations. The task requirement is not to retrieve just the full-text article or abstract, but also the evidence sentence that has the most marked evidence of the BEL statement. After retrieving the relevant citations, the abstracts and full-text articles are split into individual sentences using the Stanford Core NLP tool (27). The ranking component further ranks the individual sentences, as described in the next section.

### Ranking component

**Figure 3.**
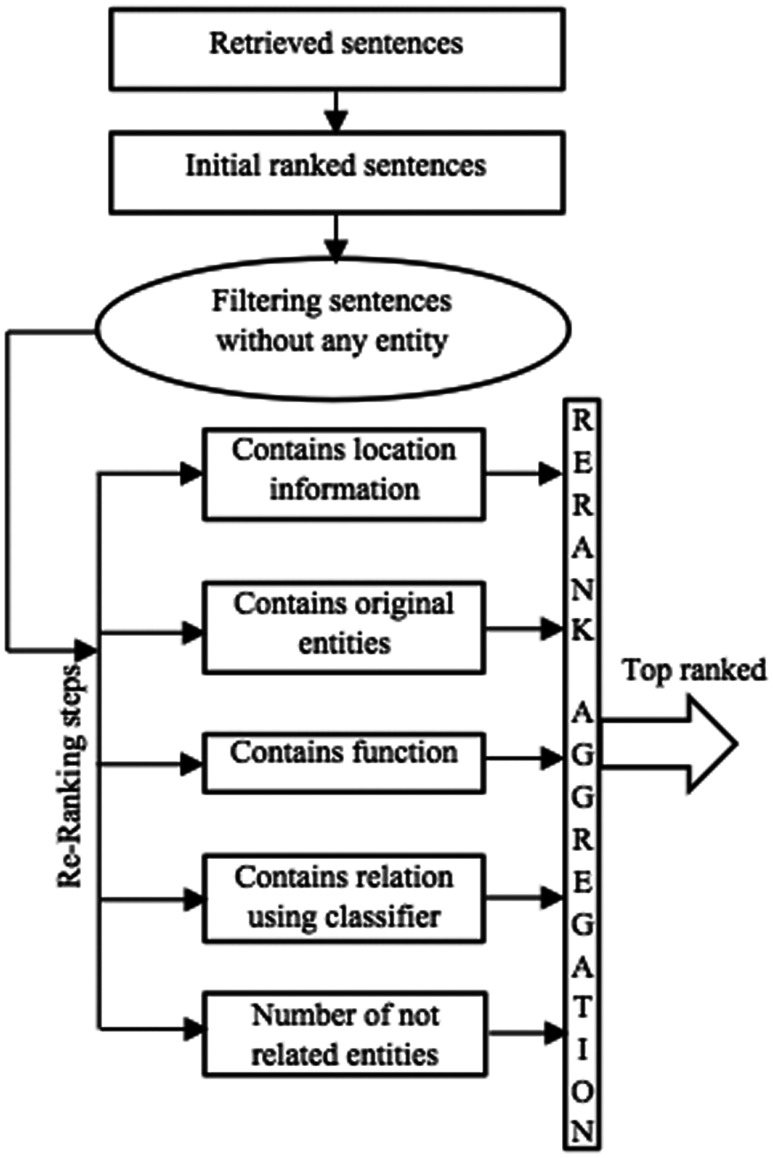
The ranking component.

Figure 3 outlines the individual steps in the ranking component of BELTracker. After retrieving the top 2000 citations (1000 from abstracts and 1,000 from full-text articles), as described in Retrieval component section the system further extracts and ranks the evidence sentences. To rank the individual sentences, the system computes the relevance, based on the number of matching elements, between the given BEL statement and the retrieved sentences. The sentences that contain more components of BEL are ranked higher. To identify the individual elements of the query within the sentences, BELTracker uses a dictionary-based approach. The likelihood of identifying functions or relationships within the sentences is lower than identifying entities, which may be partially due to the limited vocabulary to identify functions and relations. Similar to assigning differential weights to the individual components of the query, BELTracker assigns different weights to the individual elements detected within the sentences. The system assigns weights for each of the elements in the following order of preference:

1. **Entities:** The main element of a BEL statement is an entity. First and foremost, an indicator of relevance between an evidence sentence and the given BEL statement is the appearance of the BEL entities within the sentence. Therefore, the system assigns the highest weight to those sentences that contain the entities mentioned in the BEL statement. Even among the entities, higher preference is given to those sentences that exactly contain the same entities mentioned in the BEL statement when compared to the occurrence of synonyms of the entities.
2. **Elements of functions:** Certain functions, such as *protein modifications*, often contain such elements as residue modified and the residue position that occurs in the protein sequence. For example, consider the function *pmod(P,S,520).* While the presence of the word *phosphorylation* or its variants and the mention of the amino acid residue *serine* is important, the mention of position *520* in the sentence makes it extremely relevant, and it should therefore be ranked higher. The system assigns differential weights to even functional elements identified in the evidence sentences. Based on our observation of training data, we assigned such differential weights to the function elements.
3. **Functions:** Functions themselves carry less weight when compared to the presence of their elements within those functions. Nevertheless, their appearance is often more than the relation and, hence, should be weighed higher than the relation keywords.
4. **Relations:** The lowest weight is assigned to the appearance of any term related to the BEL relations due to thedifficulty of defining and identifying these terms. In addition to the appearance of any related term, we use a binary classifier to classify sentences into *increase* or *decrease* classes (the two main types of BEL relations). The result of the classifier is another factor in the ranking component. We describe the role of the classifier later in this section.

The presence of the number of irrelevant entities within the sentences dilutes the ranking of the sentence for a given query. The system filters out those sentences that do not contain all the entities (or their synonyms) mentioned in the given BEL statement. It then ranks the remaining sentences based on the number of matching elements.

### Relation classifier

To identify relationship types, we trained a binary classifier to classify sentences into two classes: *increase* and *decrease*. For training the classifier, we used the training data provided by the organizers, which contain 11 073 BEL statements extracted from 6359 sentences. We used unigrams (after removing stop words), bi-grams, entities within sentences, and part-of-speech tags of all words between the entities as features. Using 10-fold cross validation, we compared the performance of several learning models such as the Support Vector Machine (SVM), Naïve Bayes and Random Forest, on the training dataset. The class label assigned to the sentences by the classifier became another parameter in the ranking.

## Data and evaluation

The organizers provided a training dataset that consisted of 11 073 BEL statements extracted from 6359 sentences. Several BEL statements were curated based on the information from a single sentence from both abstracts and full-text articles. The training data was common to both the tasks.

For the evaluation, they provided two separate corpora for tasks 1 and 2. The test data for the second task contained 97 BEL statements. The organizers defined the following three criteria for evaluating the performance of the system (1).

1. ***Full*:** Used if the identified evidence sentence contains the complete BEL statement.
2. ***Relaxed*:** The retrieved sentence may not have all the evidence for extracting the complete BEL statement. However, it may have the necessary context and/or biological background to enable extraction of the full BEL statement.
3. ***Context*:** Even though the complete or partial BEL statement cannot be extracted from the sentence, it provides the necessary context for the BEL statement. The entities or their synonyms mentioned in the BEL statement are also identified in the sentence, but the context description (function or the links) may not accurately reflect the actual BEL statement.

The task organizers manually evaluated the results to determine the relevance of the evidence statements for a given BEL statement. They calculated the system’s precision for each criterion using Equation 1.

$$Precision=\;\frac{True\;Positive}{True\;Positive+False\;Positive}(Equation\;1)$$

In addition to precision, organizers also computed the mean average precision (MAP) (1) to evaluate the ranking quality of the system. They compared the system MAP with three alternative ranking scenarios (worst, random, and best).

1. ***Worst:***All the true positives (TPs) are ranked after all false positives (FPs).
2. ***Random:***Randomly reordered the results 2000 times and computed the average MAP for all these variants.
3. ***Best:***All TP are ranked before all FP.

## Results

Table 2 illustrates the performance of the relation classifier (used in the ranking component) against the training dataset, applying different feature sets and learning models. The results show that the Random Forest classifier obtained the highest F-measure using unigrams, part-of-speech tags, and bi-grams as features.

**Table 2:** Performance of the binary relation classifier against training data set

Model	Features	F-Measure
Naïve Bayes	Unigram	0.682
Naïve Bayes	Unigram + POS	0.711
Naïve Bayes	Unigram + POS + Bi-gram	0.714
Random Forest	Unigram	0.810
Random Forest	Unigram + POS	0.813
Random Forest	Unigram + POS + Bi-gram	0.822
SVM	Unigram	0.623
SVM	Unigram + POS	0.646
SVM	Unigram + POS + Bi-gram	0.651

This table shows the performance of our relation classifier using different feature sets and learning models. The relation classifer is used in the ranking component to classify the evidence sentences into two main BEL relations, ‘*increase*’ and ‘*decrease*’. The results show that using the combination of unigrams, part-of-speech tags, and bi-grams obtained the highest F-measure for all three learning models. Among the three models, Random Forest achieved better F-measure using different feature sets. We have highlighted the classifier with the best performance in the above table.

BELTracker, on average, retrieved 612 citations for each BEL statement, of which 222 were from abstracts and 390 from full texts. As per the task requirements, we provided, at most, the top 10 evidence sentences for the 97 BEL statements. BELTracker returned 806 sentences for 97 BEL statements at an average of 8.3 sentences per BEL statement.

Table 3 shows the performance of BELTracker under three evaluation criteria, which were provided by the organizers.

**Table 3:** BELTracker’s performance

Criteria	True positive	False positive	Precision
Full	316	490	39.20
Relaxed	429	377	53.22
Context	496	310	61.53

BELTracker performance evaluation under three criteria, full, relaxed, and context.

*Full*: if the identified sentence contains the complete BEL statement.

*Relaxed*: The retrieved sentence may have necessary context and/or biological background to enable extraction of full BEL statement.

Context: Even though the complete or partial BEL statement cannot be extracted from the sentence, it provides the necessary context for the BEL statement.

Table 4 provides the MAP of the system comparing three different scenarios: worst, random and best.

**Table 4:** Mean average precision comparison of BELTracker against baseline [1]

Criteria	BELTracker (%)	Worst (%)	Random (%)	Best (%)
Full	49.0	31.7	46.5	74.2
Relaxed	62.1	45.9	58.4	80.4
Context	68.9	55.2	65.7	83.5

Comparison of BELTracker’s ranking against three alternative ranking baseline scenarios: Worst, Random and Best, and compared MAP of our system with these scenarios.

*Worst*: All TP are ranked after all false positives.

*Random*: Randomly reordered the results 2000 times and computed the average MAP for all these variants.

*Best*: All TP are ranked before all FP.

TP, true positives, FP, false positives; MAP, mean average precision.

**Figure 4.**
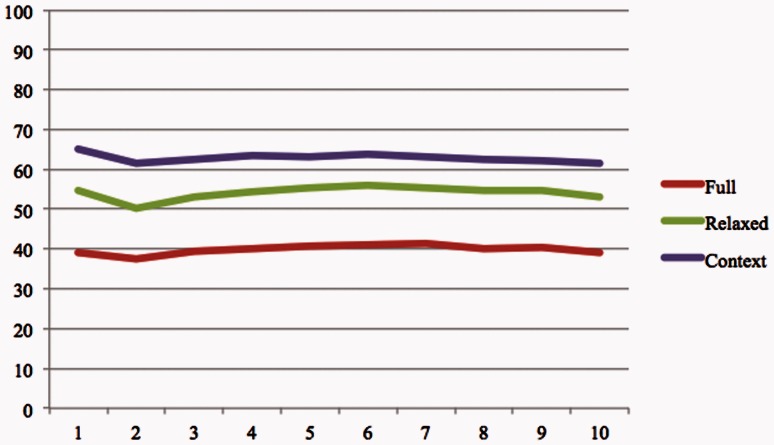
The system precision for at most K evidence sentence (*K*  =  1:10).

To analyze the impact of the ranking component, we calculated the system precision for most top K evidence sentences (*K* =  1:10). Table 5 shows the number of true positives and false positives for each run and Figure 4 illustrates the system precision.

**Table 5:** BELTracker’s performance for finding at most *K* evidence sentence

K	Full		Relaxed		Context	
	TP	FP	TP	FP	TP	FP
1	38	59	53	44	63	34
2	72	120	97	95	118	74
3	111	170	149	132	176	105
4	145	218	197	166	230	133
5	179	262	244	197	279	162
6	212	306	290	228	330	188
7	244	348	328	264	374	218
8	267	397	363	301	414	250
9	298	437	401	334	456	279
10	316	490	429	377	496	310

K, Maximum number of returned sentences for each query; TP, true Positives; FP, false positives.

## Error analysis and discussion

Out of the 97 statements in the test data, our system was able to retrieve at least one evidence sentence for 96 BEL statements. BELTracker failed to find any evidence sentence for only one BEL statement (given below):

“p(HGNC:IL1A) increases r(HGNC:DEFB4A)”

The query for the retrieval engine had the following components, *IL1A* and *DEFB4A,* with their synonyms included along with the term *increase*. Performing a similar query in PubMed resulted in only one abstract, which seemed to be relevant to the above BEL statement. Preliminary analysis indicates that the IR capabilities of BELTracker may be far limited when compared to the Entrez retrieval engine from NCBI. One way we plan to address this issue is to use the results of Entrez. In the following sections, we analyze the system performance under the *Full* and *Context* criteria.

### Full criterion

To streamline our discussion, we grouped our results into two categories:

1. Group A, where the system identified at least one evidence sentence within the top 10 that was relevant for constructing the complete BEL statement, even though they may not be among the top ranked.
2. Group B, where the system failed to identify at least one evidence sentence that is relevant for constructing the complete BEL statement.

As per the manual evaluation by the organizers, there were 72 statements that fell into group A and 27 that fell into group B.

In group A, the system ranked 53% (38/72 BEL statements) of the evidence sentences that provided evidence for extracting complete BEL statement at the top, while the remaining 47% were ranked within the top 10. This result indicates that while the system was successful in retrieving at least one fully relevant evidence sentence, its inability to rank those sentences on top of the lists (for 34 [47%] of the BEL statement) reveals the limited capability of the ranking component of the system.

We further investigated why the system was unable to retrieve any sentence that will help construct the complete BEL statement within the top 10 for group B statements. We counted the number of retrieved citations from PubMed abstracts and full texts and compared that number to group A. The average number of retrieved citations for statements was higher (650) for group A compared to group B (510). We speculate that our decision to make the entities the only mandatory component to match in the evidence sentence was a key reason for this difference. We further analyzed the performance of BELTracker by observing its trend across different entity namespaces. Table 6 lists the percentage frequency of each of the entity namespaces in the BEL statements provided for blind evaluation. From Table 6 we can infer that BELTracker failed to find any evidence sentence for 42% of the statements containing *HGNC* entities, which is higher than the other namespaces, especially when compared to *MGI* entities, which was only 19%. One possible explanation for the lower performance in finding evidence statements for humans (*HGNC*) compared to mice (MGI) may be due to the bias in existing literature towards mice (more than humans). These findings suggest that there is enough scope to further improve the ability of BELTracker to identify and rank sentences that contain evidence for extracting a complete BEL statement.

**Table 6:** Percentage frequency of entities from different namespaces in the statements with and without retrieved evidence sentences

Namespace	Percentage frequency of entities	
	Statements with evidence sentence (Group A)	Statements without evidence sentence (Group B)
HGNC (Gene)	47 (58%)	34 (42%)
MGI (Gene)	71 (81%)	16 (19%)
Gene Ontology (biological processes)	13 (81%)	3 (19%)
CHEBI	9 (81%)	2 (19%)
MESHD	8 (100%)	0

### Context criterion

The performance of BELTracker was better when evaluated for finding sentences that contain necessary context for extracting a BEL statement. First, under context criterion, the system was able to retrieve at least one relevant evidence sentence for 81 out of 97 statements, which is 9% higher than the full criterion. The system has the potential of extracting more contextual information for BEL statement curation than identifying the correct sentence to extract a complete BEL statement.

We also observed a considerable difference in the performance of the system (61% for context vs. 39% for full) during manual evaluation by organizers under the two criteria. In both Table 5 and Figure 4, we observe no substantial drop in the precision of the system among the top 10 ranked results in the increasing order of rank. This, to some extent, points to the need for sophisticated ranking approaches to appropriately rank the evidence sentences. Currently, we rely extensively on the lexical feature of the components of a BEL statement, ignoring much of the underlying semantics expressed in BEL. We firmly believe that enriching the lexical capabilities of the system with deeper semantic analysis will considerably improve the precision of the system. We plan to achieve this by integrating the information extraction capabilities [system in task 1 of BEL track (28)] into our IR workflow. As an immediate next step, we plan to process all evidence statements retrieved by our IR system through the IE engine. Based on the semantic distance between the BEL statements extracted by the IE system and the gold standard, we will be able to improve the ranking of the evidence statements. The other approach we plan to employ is integrating semantic predications extracted by a rule-based system, called SemRep (29, 30), from PubMed abstracts. Each semantic predication is a subject-relation (predicate)-object triple. *Subject* and *object* are concepts from the UMLS Metathesaurus and *predicate* is a relation from the UMLS Semantic Network. These semantic predications will allow BELTracker to take advantage of the already extracted relationship between concepts to judge further their relevance to a given BEL statement.

### Limitation

At this moment, the system employs various heuristics, based on the lexical features, to rank the evidence sentences. As pointed out in an earlier section, we plan to address this by extensively taking into account the semantic features embedded in a BEL statement in order to further improve the accuracy of the system. BELTracker has a very long response time for a given query, usually on the order of a few minutes. This slower response time needs to be addressed, given that users typically expect responses closer to a few milliseconds from IR. All the ranking methods operate at the sentence level. In the initial design, most of the pre-processing, such as sentence tokenization etc., were handled as they happened, which resulted in a very slow response from the search engine. Recently, we revamped the implementation by pre-indexing all the PubMed abstract sentences. Pre-indexing sentences considerably improved the response time of the system from a few minutes to very few seconds. We hope to improve the processing time further by pre-indexing all metadata components of BEL statements, including entities, functions, and relations.

## Conclusion

We participated in the BEL track of BioCreative V and developed an information retrieval system called BELTracker to retrieve and rank evidence sentences for a given BEL statement. Our system contains three components: (i) query translation, (ii) retrieval and (iii) ranking components. BELTracker retrieves evidence sentences from PubMed abstracts and full-text articles available at PubMed Central. Our system was able to rank at least one evidence sentence among top 10 returned sentences for 72 out of 97 BEL statements of the test set. The precision of our system under full, relaxed, and context criteria was 39, 53 and 61%, respectively. Our error analyses indicated the need to explore a more sophisticated ranking mechanism that considers semantics to improve the precision of the system. We were the only group to participate in this task.

## Funding

This work was supported by the grants from the National Institutes of Health (RO1LM11829, RO1GM102282, RO1LM11934, RO1LM11369).

*Conflict of interest*. None declared.
